# Engineering nano-drug biointerface to overcome biological barriers toward precision drug delivery

**DOI:** 10.1186/s12951-022-01605-4

**Published:** 2022-08-31

**Authors:** Saquib Waheed, Zhibin Li, Fangyingnan Zhang, Anna Chiarini, Ubaldo Armato, Jun Wu

**Affiliations:** 1 Department of Burn and Plastic Surgery, Shenzhen Institute of Translational Medicine, The First Affiliated Hospital of Shenzhen University, Shenzhen Second People’s Hospital, Shenzhen, 518035 China; 2 Department of Biomedical Engineering, School of Medicine, Shenzhen University, Shenzhen, 518060 China; 3 Human Histology & Embryology Section, Department of Surgery, Dentistry, Paediatrics & Gynaecology, University of Verona Medical School, 37134 Verona, Venetia Italy

**Keywords:** Nanoparticle, Biological barriers, Drug delivery, Nanomedicine

## Abstract

The rapid advancement of nanomedicine and nanoparticle (NP) materials presents novel solutions potentially capable of revolutionizing health care by improving efficacy, bioavailability, drug targeting, and safety. NPs are intriguing when considering medical applications because of their essential and unique qualities, including a significantly higher surface to mass ratio, quantum properties, and the potential to adsorb and transport drugs and other compounds. However, NPs must overcome or navigate several biological barriers of the human body to successfully deliver drugs at precise locations. Engineering the drug carrier biointerface can help overcome the main biological barriers and optimize the drug delivery in a more personalized manner. This review discusses the significant heterogeneous biological delivery barriers and how biointerface engineering can promote drug carriers to prevail over hurdles and navigate in a more personalized manner, thus ushering in the era of Precision Medicine. We also summarize the nanomedicines' current advantages and disadvantages in drug administration, from natural/synthetic sources to clinical applications. Additionally, we explore the innovative NP designs used in both non-personalized and customized applications as well as how they can attain a precise therapeutic strategy.

## Introduction

Nanotechnology is a broad horizon of emerging technologies that can be used to enhance medical treatments. Nanoparticles (NPs) are solid particles or particulate dispersions that contain nanocapsules and nanospheres and are usually produced using polymerization techniques and preformed polymers [1]. Engineered nanoparticles (ENPs) have tremendous potential for increasing disease detection and therapy specificity. Nanomedicine is a multidisciplinary field that encompasses the fusion of biotechnology, nanotechnology, and information technology, resulting in many applications, including drug delivery and in vivo treatments, diagnostic devices, molecular imaging, biomarkers, regenerative medicine, and biosensors, etc. [2]. Nanotechnology offers multiple benefits to overcome the constraints associated with conventional drug administration methods through site-specific and target-oriented conveyances. NPs have shown great potential to enhance the solubility and stability of encapsulated cargos, improve cell uptake, and lengthen circulation duration, which altogether helps increase safety and effectiveness [3, 4]. As a result of these factors, researches on NPs have been widely conducted, yielding encouraging findings in vitro and in vivo (small animal models) [5]. However, delivering a drug to its targeted location is still particularly challenging regardless of these apparent merits.

Recent research showed that only 1% of the intravenously delivered NPs reach their intended target sites [6]. Efforts have been made to increase drug carrier targeting efficiency by attaching cell receptor-targeted ligands to the particle surface [7]. Although this is a significant step forward in achieving targeted drug delivery, the drug carrier must have to overcome various biological obstacles throughout the body to reach the specific target of interest. Even after arriving at their sites of action, prior to the release of their cargo and clearance, NPs must effectively penetrate through the cell membrane and navigate intracellular compartment networks to get to their subcellular mark. Apart from carrier biodistribution, various other impediments require proper overcoming. Despite the comprehensive research prompted by world-renown scientists around the globe, the number of nanomedicines approved by the Food and Drug Administration (FDA) is far less than anticipated, in part due to the translational gaps between animal model species and human trials outcomes, which are not as satisfying as expected [5, 8]. These gaps stem from a persisting lack of knowledge of the physiological and pathological differences between animal models and humans, particularly concerning the mechanisms by which these variations shape nanomedicines' distribution and effectiveness inside the body [9]. Clinical translation of therapeutics is hampered by several factors, of which species differences are not the least ones. Also, patient heterogeneity can hinder nanomedicines' efficacy as currently, only a limited number of clinical trials are available on nanomedicines interactions with stratified patient groups. As a result, only a few nanomedicines are presently suggested as first-line alternative treatments, and many of them only benefit a small proportion of patients [10]. This underappreciated variability in the biological foundations of diseases and across patients partially affects NPs effectiveness by altering the development, shape, and physiology of the pathology-affected tissue.

Considering the vast number of publications and the speed at which data is updated, this study explores the progress and applications of Nanotechnology in the realm of Nanomedicine through a broad, comprehensive overview of the most commonly investigated NPs. Because we expect that Precision Medicine therapies will profoundly affect precision NPs in the near future, we discuss critical biological barriers to targeted therapies involving precision drug delivery and their clinical transition to improve patient-specific therapeutic responses. Additionally, we discuss the challenges and opportunities associated with the most promising and demanding applications of NPs in modern Nanomedicine.

## Classes of NPs

### Lipid-based NPs

**Fig. 1:**
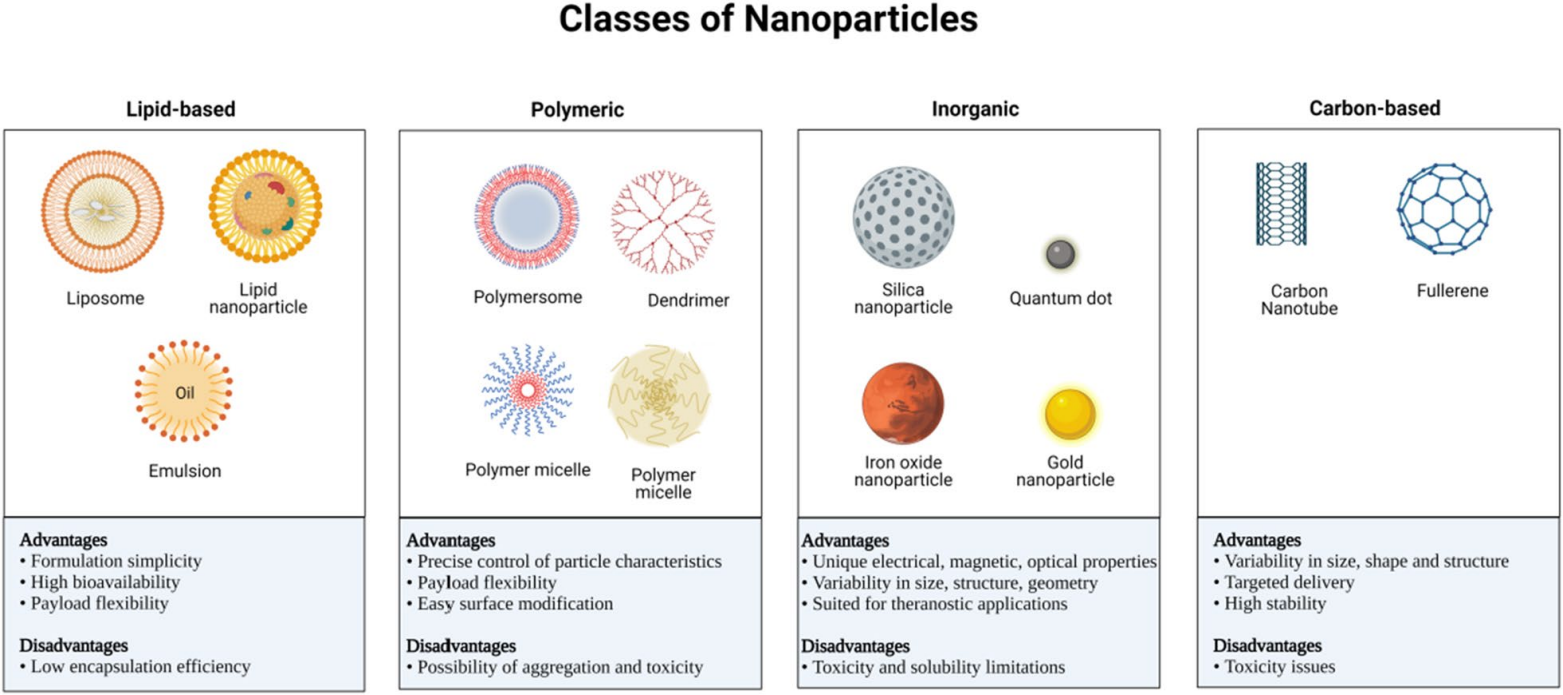
The diverse types of nanoparticles. Nanoparticles (NPs) could be classified into four main categories according to size, shape, and physicochemical properties. Each class of NPs includes several subclasses, some of which are highlighted here. Each class has numerous advantages and disadvantages regarding conveyed cargo, delivery, and patient responses

The past decade has witnessed significant attention from scientists toward the Lipid-based NPs (LBNPs), which, due to their biocompatibility, were even considered “nanosafe” carriers. LBNPs present various shapes and sizes, but the most common are vesicle-like spherical platforms made by at least one lipid bilayer surrounding an aqueous interior compartment (Fig. 1). LBNPs can transport large payloads and exhibit various physicochemical properties that can be manipulated to alter their biological features [11]. Such vesicles can upload hydrophobic and hydrophilic pharmaceuticals [12, 13], including vaccines [14], insulin [15], siRNAs [16], proteins [17], and enzymes [18]. They can also be administered intravenously, orally, or transdermally to treat disorders including Alzheimer's disease, cancer, and diabetes [19, 20]. For these reasons, LBNPs are common to most FDA-approved nanomedicines [10, 21].

Since liposomes were first characterized by Alec Bangham in 1960, liposomes sparked attention for several decades, most notably when Doxil® was licensed by the FDA and utilized in clinical trials [20]. Liposomes are the largest category of LBNPs, primarily made of phospholipids, and can take on unilamellar or multilamellar vesicular shapes [22]. Liposomes are remarkable drug delivery vehicles as their composition resembles cell membranes. Additionally, they are also biocompatible and biodegradable and can improve drug stability and biodistribution. Generally, NPs stability in vivo and in vitro is mainly affected by their shapes, sizes, surface charges, surface modifications, and preparation methods; these characteristics can be modified during their synthesis [23]. Liposomes are frequently modified (with polymers or ligands) to improve circulation and distribution, making them even more suitable for clinical usage [24, 25]. Several liposome-based biomedical applications are either in clinical trials or soon on the market [26–28]. A recent study examined the ability of Pluronic F127 (PF127)- and PEGylated liposomes to penetrate a pathological mucus obtained from chronic obstructive pulmonary disease (COPD). The PEG-liposomes penetrated the mucus barrier effectively after 27 h of incubation [29]. Similarly, nanoscale diagnostic agents can be enclosed within theranostic liposomes for imaging purposes, wherein the therapeutic agent can be encapsulated in the core or incorporated in the lipophilic bilayer shell [30–32]. For example, to improve magnetic resonance imaging (MRI), superparamagnetic iron oxides (SPIONs) can be coated with a lipid layer or encapsulation of gadolinium(III) chelates in the aqueous core of liposomes. Likewise, Li et al*.* demonstrated the multifunctional liposome containing lipidized gadolinium-DOTA lipids not only could be used as an MRI imaging probe but also suitable for near-infrared fluorescence imaging relying on the lipidized near-infrared dyes [33]. Additionally, LBNPs are currently being highlighted as an instrumental component of the COVID-19 mRNA vaccines, in which they play a major role in protecting and transporting mRNA into the cells [14, 34]. With the successful use of LNPs as a delivery vector for the COVID-19 mRNA vaccines, the research horizons for mRNA vaccines will likely be broadened.

Lipid nanoparticles (LNPs) are another prominent subgroup of LBNPs and often employed for nucleic acid delivery. They differ from standard liposomes because of their particulate core, and they produce micellar structures that can be changed depending on the preparation methods and formulation parameters [35]. LNPs are usually made of four key components: phospholipids assisting in particle structure; cholesterol aiding membrane fusion and stability; cationic or ionic lipids combining with negatively charged genetic material to promote endosomal escape; and PEGylated lipids improving circulatory conveyance and stability [36]. LNPs have increased their relevance to applications involving customized genetic treatments due to their efficacy in nucleic acid delivery, as well as to their small size, ease of synthesis, and stability in the serum [17]. In order to administer these nucleic acid-based therapeutics, ionizable LNPs are a robust delivery platform because they have a physiological near-neutral pH. However, once endocytosed, they become fully charged in the acidic endosomal compartments, facilitating their endosomal escape for intracellular drug delivery [37, 38]. Despite these benefits, LNP systems might be hampered by poor drug loading and biodistribution, resulting in their significant uptake by the liver and spleen [21].

### Polymeric NPs

Polymeric nanoparticles (PNPs) are colloidal systems synthesized from either natural or synthetic polymers and classified into nanocapsules and nanospheres. PNPs have been extensively explored in Nanomedicine due to their surface modification capabilities, they may be synthesized to finely control NPs characteristics. For example, drugs can be loaded inside the PNP's core cavity or chemically bonded to the NP's surface. PNPs are also excellent vehicles appropriate for co-delivery applications because they can carry both hydrophobic and hydrophilic drugs and a wide range of cargos with different molecular weights [39–41]. To date, PNP-based materials have been increasingly employed in nanocomposites, cancer medication delivery, and photovoltaic applications. The smaller particle size of PNPs is one of the key aspects that makes them different from their counterparts. PNPs could be further enhanced and precisely controlled due to their specific qualities to attain desirable attributes for improved biocompatibility and bioavailability [42, 43].

Nanospheres are slightly bigger colloidal NPs (indeed, their shape is not invariably spherical); nanocapsules, on the other hand, resemble a vesicular system composed of a polymeric shell. Within these two broad groups, NPs are further classified according to their morphology into polymersomes, micelles, and dendrimers. For instance, polymersomes are artificial vesicles that have copolymer membranes endowed with amphiphilic blocks. They are similar to liposomes in that they typically are locally responsive. Still, they have better stability and a higher cargo retention efficiency [44], making them serve as valuable vehicles for delivering drugs to the cytosol [45].

Poly(dimethylsiloxane) (PDMS) and poly(ethylene glycol) (PEG) are two smart polymers that are often copolymerized as polymeric micelles for medical applications. Polymeric micelles are nanoscale drug delivery systems. They are responsive block copolymers self-assembled into nanospheres with a hydrophobic core and a hydrophilic coating that protects an aqueous drug cargo and promotes circulation. Polymeric micelles are generally flexible carriers of a variety of payloads and therapeutics and have been utilized to deliver cancer therapies in clinical studies [46]. In a doxorubicin (DOX)-resistant ovarian cancer cell spheroid model, PEG-phosphatidylethanolamine (PEG2000-PE) micelles loaded with DOX and targeting with anti-nucleosome mAb 2C5 were found to be effective. Compared to free DOX and non-targeted DOX micelles, the 2C5 targeted DOX micelles showed improved uptake and penetration abilities and finally induced greater cell death [47]. Similarly, in another case, Hu et al*.* proposed a nanoplatform with paclitaxel (PTX) encapsulated within a triblock Polycaprolactone (PCL)-PEG-PCL copolymer that confirmed sustained drug release in combination with circadian chrono modulated chemotherapy [48]. Moreover, Hong et al*.* obtained image-guided polymeric micelles containing DOX and SPIONs NPs in a folate-conjugated PEG-b-PCL copolymer [49]. These studies offer superior treatment efficacy and real-time tracking of the drug delivery system in vivo, which is crucial for designing more promising drug delivery systems.

Dendrimers are highly branched polymers with irregular or complex 3D structures. Due to their highly controlled size, shape, mass, and surface modifications can incorporate various therapeutics to generate physiologically active drug conjugates. On the outside, dendrimers own active functional groups that allow external contrast agents or biomolecules to be conjugated while the therapeutics can be loaded inside the shell. Dendrimers can carry a variety of payloads, although they are most typically used to deliver nucleic acids and other smaller compounds [50]. The use of charged Polyamidoamine (PAMAM) dendrimers is common as they are a prime example of these versatile applications. Numerous compounds derived from dendrimers are now being evaluated in clinical trials, including transfection agents, contrast agents, and topical gels [50, 51].

In general, PNPs are excellent candidates for drug delivery due to their biodegradability, water-solubility, and biomimetic properties. Their surface can be readily modified to facilitate further targeting [30], enabling them to transport genetic material, therapeutics, and proteins to specific organs or tissues. Therefore, PNPs are valuable tools in gene therapy, diagnostics, and the successful treatment of various diseases. Regardless of the benefits, PNPs entail a higher risk of toxicity and particle aggregation. Although a limited number of polymeric nanomedicines have been authorized by the FDA and are already being used in clinics, many new generations of polymeric nanocarriers have already been preclinically tested and are currently evaluated in clinical trials [10].

### Inorganic NPs

Recent breakthroughs in Nanotechnology have permitted the use of several inorganic compounds, including iron, gold, and silica, to synthesize NPs for diverse drug delivery applications (Fig. 1). Inorganic NPs can be designed and synthesized to achieve a broad range of shapes, sizes, and geometries. The most thoroughly studied gold NPs (AuNPs) are fabricated in various shapes, including nanoshells, nanospheres, nanocages, and nanorods [52]. Furthermore, due to the qualities of the constituent materials, inorganic NPs show distinct optical, electronic, magnetic, and thermo-physical or mechanical properties. For instance, the photothermal characteristics of AuNPs are attributed to the presence of delocalized free electrons that continuously oscillate at a frequency depending on the particle's size, shape, and structure [53]. Additionally, functionalization can easily modify AuNPs to enhance their characteristics and ability to transport drugs, thus making them ideal for biomedical and biological applications [52].

Iron oxide NPs (IONPs), which hold a privileged position among the bulk of FDA-approved potential inorganic nanomedicines, is another extensively studied material for inorganic NP synthesis [54]. Magnetic IONPs are composed of maghemite (γ-Fe_2_O_3_) and magnetite (Fe_3_O_4_) and show superparamagnetic characteristics at particular sizes (diameters ranging from 1 and 100 nm). They have demonstrated effectiveness as thermal-based therapeutics, contrast agents, and drug-delivery systems [55]. Many researchers have used magnetic hyperthermia in combination with traditional methods to treat various types of cancers following the first clinical application of magnetic hyperthermia in 2011 [56, 57]. In recent research, SPIONs have been packaged into exosomes derived from mesenchymal stem cells. These exosomes are efficiently internalized by tumor cells, supporting remote targeting and ablation of tumor cells. Meanwhile, SPIONs coated with lipids can also be a carrier for poorly soluble drugs in water. According to a previous report by Ong et al*.* the therapeutic efficacy of water-insoluble methotrexate (MTX) against MDA-MB-231 breast cancer cells is prominently enhanced when encapsulated in the SPIONs lipid-based homeostasis system [58]. In this respect, an effective multimodal treatment regimen was developed with strong colloidal stability and bio- and hemocompatibility. In addition to killing breast cancer cells, hyperthermia helped the controlled release of drugs [59]. Other types of inorganic NPs often used include mesoporous silica and calcium phosphate NPs, both of which have been effective deliverers of genes and drugs [60, 61].

Overall, inorganic NPs are perfectly qualified candidates for drug delivery, magnetic resonance imaging, photothermal therapies, and diagnostic applications, owing to their unique radioactive, magnetic, and optical properties. The majority of inorganic NPs own strong stability and biocompatibility, thus fulfilling niche applications that need qualities that organic materials cannot provide. However, the intrinsic toxicity and low solubility restrict their practical applicability, particularly in the case of heavy-metal composites [62].

### Carbon-based NPs

Carbon-based nanomaterials are a class of their own, entirely composed of carbon atoms, such as graphene, fullerene, and carbon nanotubes. These materials have historically been overlooked for biological applications due to their stiff structure and poor water solubility. After a series of particular modifications, the unique physicochemical properties of these materials became manifest [63]. For instance, the graphene oxide-quaternary ammonium salt nanocomposite exhibited significant synergistic antibacterial activity and enhanced wound healing by boosting re-epithelialization and granulation tissue development [64]. Recently, a study investigated the feasibility of carbon nanotubes as radiosensitizers and reported a surprising effect, i.e., the suppression of prostate cancer cells with no need for an antihormone treatment [65]. Carbon nanotubes could be used even as de novo antiviral nanomedicine intermediates to prevent SARS-CoV-2 [66]. Additionally, carbon NPs can be functionalized and employed in a variety of applications. The medical use of C60 is primarily due to its ability to be modified by adding side-chains with functional groups, which enhance its biocompatibility and thus enable its use in the treatment of various diseases [67]. Interestingly, C60 has shown exceptional effectiveness in regulating allergic responses for prospective therapeutic applications.

Even with these advances, there are some drawbacks and toxicity limitations related to carbon-based NPs. The carbon's surface charge determines the toxicity level; cationic surfaces are more hazardous than anionic or neutral surfaces. Smaller particles (< 50 nm) are more functional as they possess a great capacity to infiltrate the cells [68]. Anyway, larger-sized particles (> 100 nm) can accumulate but not properly diffuse within some organs, while smaller particles can be taken up by macrophages and transferred to local lymph nodes. According to experimental studies conducted with HeLa cells, spherical mesoporous silica NPs with a diameter of 50 nm displayed the greatest cellular uptake [69]. Moreover, a study examining 40–50 nm AuNPs in SKBR-3 cells reported the highest cellular uptake [70]. Similar results were noted when the core was changed from gold to silver. In vitro studies consistently demonstrated maximum uptake within the 10–60 nm range, irrespective of their surface charge or composition [9, 12, 22, 33, 34]. However, it is also important to consider the type and location of the targeted tissue when determining the optimal size of a NPs. Different studies on animal models have demonstrated that NPs administration into the lungs caused airway inflammation characterized by a cellular infiltration comprised of neutrophils and macrophages and the generation of pro-inflammatory cytokines [71, 72]. Similarly, two separate studies demonstrated that inhaled multiwalled carbon nanotubes would increase airway fibrosis in mice and remodeling and increased production of epithelium-derived innate cytokines with allergic asthma [73, 74]. Notably, the inhalation of drug nanoformulations has caused various lung disorders in humans, including asthma, cystic fibrosis, tuberculosis, and lung cancer [73–77]. The unwanted interactions between carbon NPs and the immune system result in immunomodulation, increasing the risk of infections.

In summary, various nanotechnology-based drug delivery systems discussed above have brought about revolutionary changes in drug delivery as well as the total medical service system. Nanotechnology can provide better insights into the molecular basis of disease. However, recent health risk evidence limits their use in the pharmaceutical industry, such as drug toxicity and particle aggregation are the two main issues associated with polymeric NPs. While inorganic NPs and carbon-based NPs have a similar dilemma. Despite current research efforts, adequate data and guidelines are still lacking regarding the safe use of nanotechnology-based devices and materials. Extensive research in this field is required to improve NP-based therapies by enhancing efficacy and reducing side effects.

## Nanoparticle-based precision drug delivery

Precision drug delivery can enhance clinical treatment methods and alleviate some of the constraints associated with current medicines. For instance, biomarkers and concomitant diagnostics have been gold standards in cancer diagnosis and therapy because most nanomedicines fail to yield favorable outcomes in unstratified studies [78]. Patient stratification is crucial to the clinical development of precision cancer therapies, but NP-based studies are presently undertaken in unstratified patient populations [79]. This is expected to change in the near future as the value of stratification is duly recognized, allowing the design of specific NPs for well-defined patient populations. As with NPs, stratified patient groups can help speed up the clinical trial process by ensuring that all the selected patients respond uniformly to a specific treatment. Furthermore, NPs can help patients qualify for Precision Medicine therapy by minimizing limitations such as comorbidities or diverse biological barriers. NPs tailored to particular patient groups have the potential to overcome present constraints in drug delivery, absorption, and metabolism, therefore qualifying more patients to benefit from Precision Medicine [80].

In the past few years, several studies have revealed that ENPs can cross various barriers. What factors influence ENPs barrier crossing efficiency is a question we need to address. According to earlier studies, various biological barriers, including microenvironmental, cellular, and intracellular barriers, impact the crossing effectiveness of ENPs [81–85].

## Biological barriers

Barriers to drug delivery restrict the effectiveness of nanotherapeutics in diseases ranging from cancer to inflammation. To achieve a successful biodistribution and drug delivery, NPs must overcome physiological and biological barriers such as shear pressure, protein adsorption, and quick clearance. [86]. These barriers are often changed in disease states, making them more challenging to overcome via a conventional, one-size-fits-all strategy [87, 88]. Such changes in biological barriers occur at the systemic, microenvironmental, and cellular levels and also vary on a patient-to-patient basis, making them difficult to identify and characterize extensively. Identifying the biological barriers that patients confront on a general level as well as on a case-by-case basis enables the development of the best ENP platforms. Site-specific drug delivery will remain an elusive target until nanocarrier design has addressed the majority of the biological barriers met upon administration. Although Nanomedicine and nanodelivery systems are emerging fields, overcoming these barriers and incorporating unique design elements will lead to the developing of a new generation of nanotherapeutics, marking a change in basic assumptions about NP-based drug delivery.

### Barriers to delivery faced by systemic administration

The barriers that NPs confront may vary depending on the mode of administration and type and progression of the disease. While local drug delivery approaches allow NPs to overcome some of the barriers associated with systemic drug distribution, they sometimes include more invasive procedures and complex techniques that impose further constraints. Additionally, local delivery may primarily benefit diseases within established and accessible areas of pathology, such as solid malignancies or traumatic injuries. Hence, systemic administration is so far prevalent in NP applications.

#### Circulation time, structural stability, and clearance in vivo

**Fig. 2:**
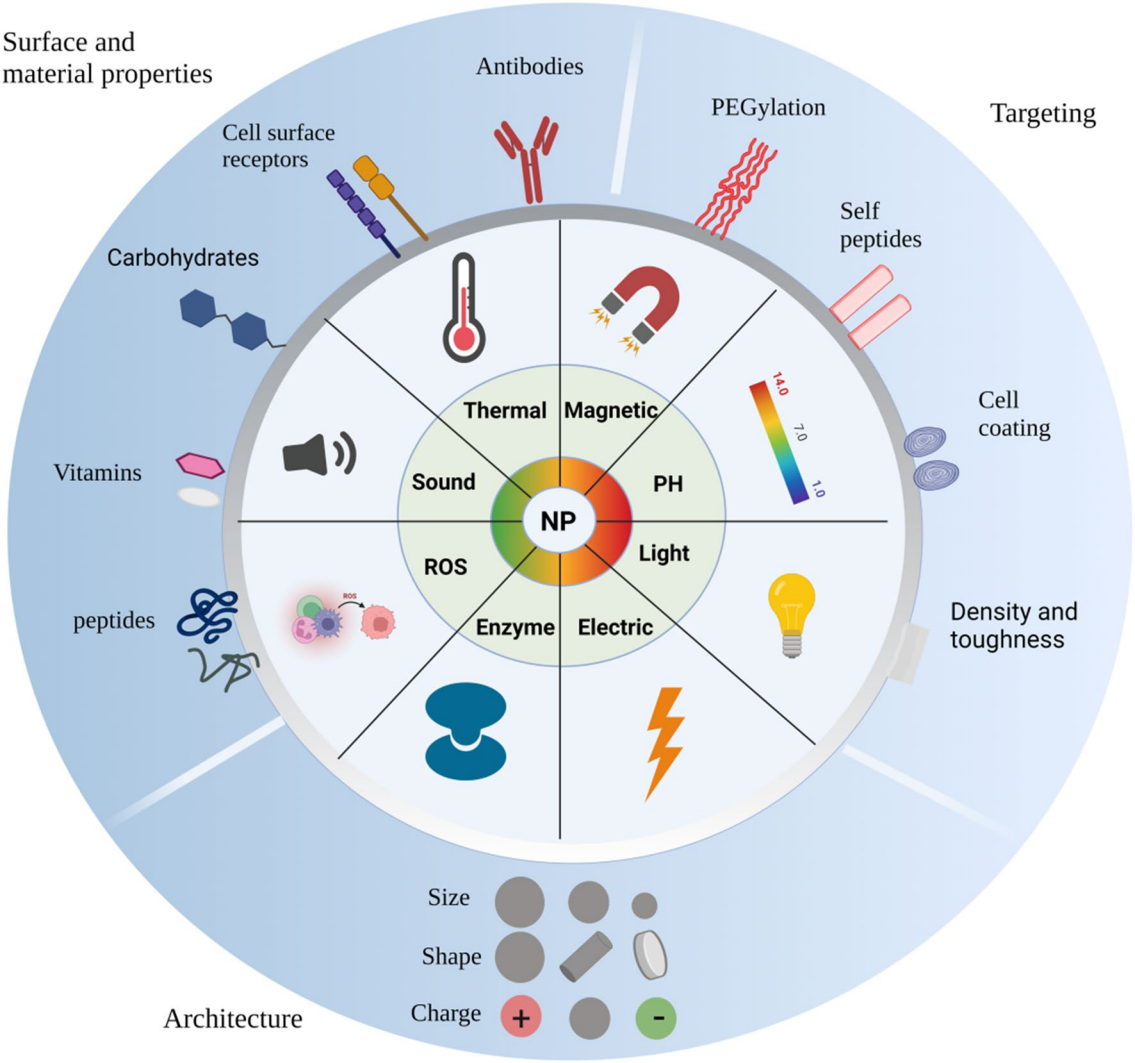
Illustration of various strategies used to develop smart nanoparticles for precision drug delivery. Material and surface properties, architecture, and targeting responsiveness are characteristics of nanoparticles (NPs) that could be intelligently modified to customize the platform for a particular application. Altogether combinations of these characteristics result in an almost infinite number of NPs features and platforms

While in circulation, NPs stability and distribution can be reduced by various factors such as blood flow, excretion, coronas, and phagocytic cells. The physicochemical features of the NP platform determine the precise effects of each of these factors (Fig. 2). This has led to formulating general design concepts to manipulate such parameters to obtain desirable outcomes. For example, NPs with a diameter smaller than 10 nm were demonstrated to be promptly excreted through the kidneys, while if not appropriately designed, NPs with a diameter greater than 200 nm risk activating the complement system [89]. Additionally, several NP formulations use PEGs as a stealth coating to minimize immediate excretion (Fig. 2). PEGylation prolongs the NPs circulation time by altering their size and solubility while protecting the NPs surface from enzymes and antibodies that could cause degradation, secretion, and rapid clearance. Various conjugation strategies were discussed by Veronese et al., along with the critical parameters of PEG structure and molecular weight (MW) required to achieve optimal efficacy of PEG-conjugated drugs [90, 91]. However, this physical barrier does not totally prevent macrophages or other immune system cells from recognizing PEGylated NPs. Furthermore, sometimes PEGylation can induce the development of anti-PEG antibodies, which might result in the rapid elimination of PEGylated NPs, when present in excess concentrations [92]. Clinical investigations have also demonstrated that these anti-PEG antibodies may be detected in individuals who have been exposed to PEG in ways other than through PEGylated medications, suggesting that even the first administered dose of PEGylated NPs may not circulate for an extended period of time in all patients [93].

Surface modification approaches enable NPs to circumvent the recognition and clearance mechanisms that might otherwise result in rapid instability and degradation. Various NP design strategies that focus primarily on stability can overcome such issues. NPs stability strongly depends on how their components interact within the environment, as lipid- and polymer-based NPs are particularly prone to unstable and aggregate during circulation and storage. Thus, to increase the stability of these softer NPs, excipients including cholesterol, helper lipids, and PEGylated lipids [23] can be incorporated in LBNPs, whereas cross-linking techniques are apt for polymer-based NPs [94, 95]. Many NPs are lyophilized for storage and transportation to increase their stability. However, this strategy does not alter the stability of NPs once administered [96]. NPs rely on a delicate balance between stability and degradation to effectively release the drug they convey, and this balance must be taken into consideration while designing the NPs. For example, the use of chitosan NPs in nanomedicine, biomedical engineering, as well as the development of novel therapeutic drug release systems with increased bioavailability, sensitivity, and specificity, has increased rapidly in recent years. In addition, chitosan stability is an important factor for its consideration in pharmaceuticals applications. Chitosan-based NPs are usually stable at neutral pH and low temperatures (2–8 ºC). However, at a slighty acidic pH or high temperature (37–50 ºC), NPs start to degrade and release the drug [97, 98]. Similarly, Poly(*N*-isopropylacrylamide) (PNIPAAm) is a thermo-sensitive polymer. PNIPAAm exhibits a reversible thermo-responsive phase transition at a lower critical solution temperature (LCST). PNIPAAm-coated NPs are stable below the LCST; however, if the temperature exceeds the specific LCST, NPs will start to degrade and release the drug [99, 100]. Once administered, NPs have to encounter varying flow rates in the bloodstream, resulting in shear stress, potentially damaging for the platforms or their payloads, and inhibiting NPs extravasation [101]. These fluid pressures can strip NPs of their surface coatings and prevent them from localizing to vessel walls [101, 102]. Larger micro-particles are more likely to adhere to vessel walls, while non-spherical particles marginate better [102]. Even after vascular localization, architecture-dependent drag forces caused by blood flow may rip NPs from endothelial cell membranes if the NPs lack an adequate binding affinity for the latter [103]. As a result, in vascular pathologies, the frequently altered hemodynamics (due to stenosis and hypertension) experienced by systemically administered NPs significantly impact NPs distribution and delivery [104, 105].

The physicochemical properties of NPs can affect their clearance from the circulation, although the interactions with the reticuloendothelial system (RES) or mononuclear phagocytic system (MPS) are more common events. Phagocytes (primarily macrophages), monocytes, and dendritic cells are involved in RES and MPS, taking up NPs and depositing them in the spleen and liver [106, 107]. This clearance occurs more rapidly in the case of rigid or stiff NPs. In general, cationic NPs are the earliest to be cleared [108], followed by anionic NPs, while slightly negative and neutral NPs have the most prolonged circulation half-lives.

Surface charge plays a significant role in determining cellular uptake efficiency and mechanism, as well as the in vivo fate of NPs [109–111]. The optimal surface charges (e.g., positive, negative, or neutral) and charge density should be carefully considered to prolong the NPs circulation time and minimize nonspecific clearance. Several NP designs incorporate surface modifications to limit clearance, such as PEG or cell membrane coatings to evade recognition and clearance by phagocytic cells [89]. Xiao et al*.* demonstrated the effect of surface charge on in vivo biodistribution of PEG-oligocholic acid-based micellar NPs [111]. In the case of highly positively charged NPs, liver uptake was very high, which may be attributed to active phagocytosis by macrophages. Conversely, liver uptake of slightly negatively charged NPs was low [111]. The phenomenon could be explained by the electrostatic interaction between the positive charge on the surface of NPs and the negative charge on the membrane of macrophages, which facilitates the internalization of NPs. It has been proposed to introduce a slight negative charge to the NPs surface to reduce the undesirable clearance by the RES. Quach et al. studied the effect of different PEG MWs (1, 2, 5, and 10 kDa) conjugated to AuNPs on their phagocytosis, revealing a larger PEG MW reduced macrophage recognition of the conjugated NPs. This effect can be explained by an increase in NP hydrophilicity that was associated with an increase in PEG molecular weight, so lowering complement protein absorption and thereby restricting complement system activation [112]. However, raising the PEG MW further might have the opposite effect.

Alongside clearance, the interactions between NPs and MPS can result in toxicity since these cells initiate immunological responses resulting in the production of tumor necrosis factors, interleukins, and interferons, all of which trigger inflammation and/or tissue damage [113]. The size, shape, and surface properties of NPs significantly affect the kind and amplitude of the immunological responses they promote (Fig. 2). Xu et al. conduct a comparative investigation to determine the tumor-targeting efficacy of ligand-modified NPs with diameters of 3 and 30 nm, respectively. The results indicated that functionalizing NPs with ligands for tumor targeting increased tumor penetration and targeting efficiency for NPs smaller than 3 nm, whereas this was not the case for NPs of 30 nm in size [114]. For instance, in a mouse ovalbumin model, spherical NPs generate a cell-mediated T-helper-1 response, micrometre-length rods induce a cell-mediated T-helper-2 response, and overall the former NPs trigger a more intense immune response than the latter ones [115].

Additionally, the uptake by phagocytic cells is directly linked to the NPs shape and aspect ratio: the uptake of triangular and rod-shaped NPs is more prominent than that of spherical or star-shaped NPs. Besides, rod-shaped NPs are more effective inflammation triggers in macrophages [116]. Similarly, in clinical studies, some PEGylated NPs sparked inflammation and produced severe allergic responses or anaphylaxis in a small proportion of individuals [117, 118]. These immunological responses to NPs modifications can cause inflammation and other undesirable side effects, suggesting the critical need to tailor NP design to mitigate these risks [119].

#### Physiological barriers

**Fig. 3:**
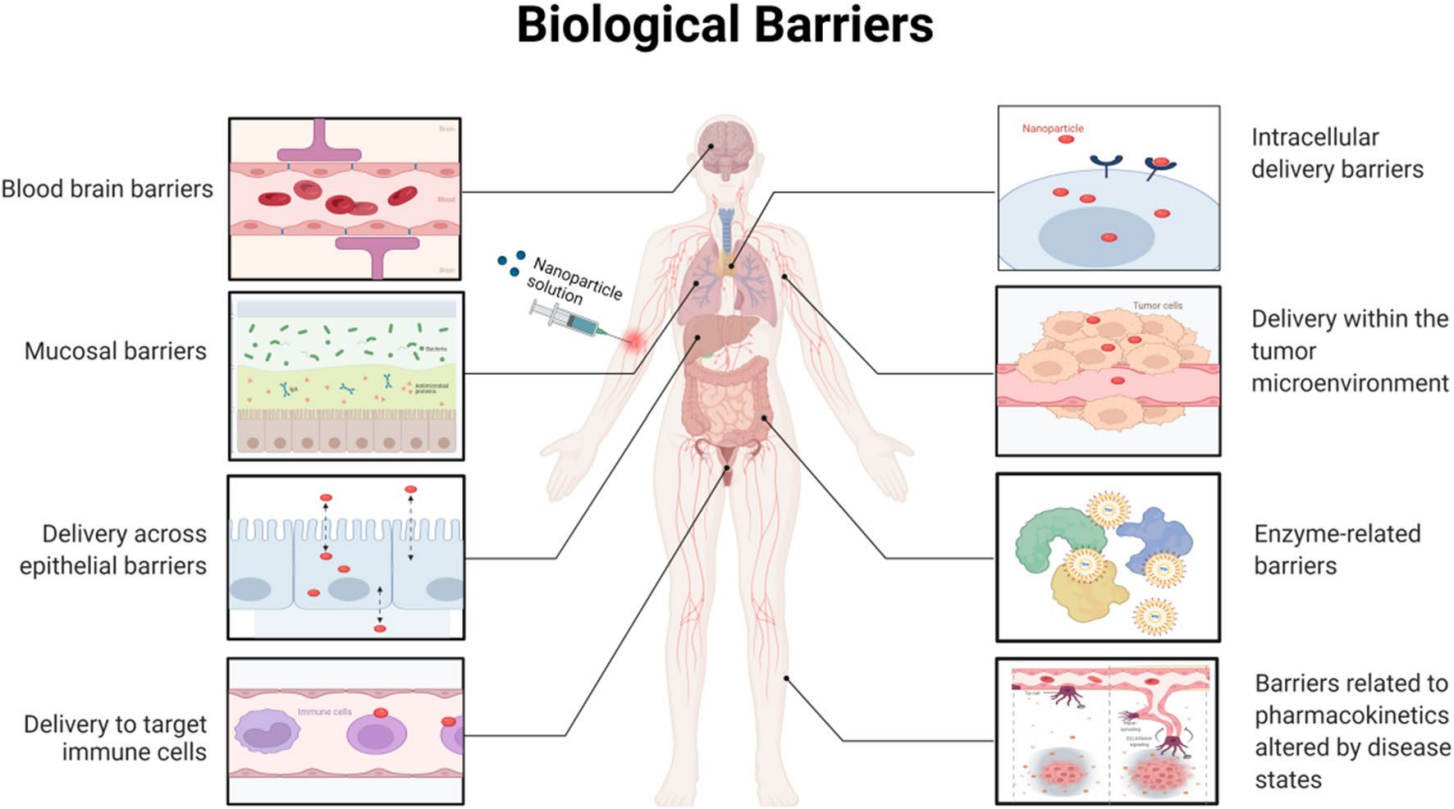
Highlighting some of the sequential biological barriers that nanoparticles (NPs) must overcome to achieve precision drug delivery. As discussed in this review, smarter NP designs that optimize delivery can significantly improve the effectiveness of precision medicines, hence expediting their clinical translation

NPs must overcome various physiological barriers to reach their targets and effectively release the drugs they convey. The frequently discussed physical barriers to NPs distribution are the tight junctions among endothelial cells (ECs) and between ECs and epithelial cells of the gastrointestinal tract, as well as microglial cells and pericytes of the blood–brain barrier (BBB) (following oral and intravenous administration, respectively) (Fig. 3). To penetrate the central nervous system (CNS), NPs need to be taken up by BBB ECs via receptor-mediated endocytosis and exocytosed on the opposite side [106, 120]. Receptor-mediated transcytosis is an efficient method of delivering drugs into the brain or infiltrating tumor tissues [121, 122]. The transporter's variability on the plasma membranes of ECs complicates BBB's crossing. However, specific transporters, such as glucose transporters, are persistently overexpressed at the BBB. Certain common targets, such as the vascular cell adhesion molecule (VCAM), can enhance NPs trafficking through the BBB [123]. These transporters and VCAM have the potential to be used to deliver NPs into the brain. Other investigated targeting options, such as the transferrin receptor, have theoretical benefits over different transporter types but have yet to prove their clinical usefulness [124]. However, only 5% of a systemically delivered NPs dosage reaches the CNS, and of that even less than 5% reaches the targeted cells [125]. Recent research on AuNPs that crossed the BBB proved that the NPs corona composition had been changed after the crossing but had remained stable. Hence, understanding the corona-altering mechanisms might help develop future CNS targeting approaches. The BBB is the main barrier to systemically administered NPs penetrating CNS tissues. Overall, to reach the brain, the intranasal administration of NPs is becoming more and more popular, as it bypasses the BBB and eliminates many of the drawbacks of systemic administration [126]. However, the intranasal route poses significant challenges due to its limited dosage volume and to variables associated with patients' mucosal congestion and amount of mucus [127]. In this respect, oral administration is the most extensively used and approved method of drug administration, however, the gastrointestinal system also places multiple barriers to NPs [128]. In the intestinal lumen, P-glycoproteins and concentration gradients limit NPs passive diffusion. Conversely, certain NPs characteristics may ease the transit through the gastrointestinal wall. For instance, smaller, negatively charged silica NPs improved intestinal permeability by opening tight junctions, thereby reducing the need for cellular absorption to affect the transport across the epithelial barrier [129].

However, size is still a concern for platforms that depend on endocytosis and exocytosis to penetrate the gastrointestinal wall. For instance, the increased surface area of polymeric NPs (compared to soluble drugs) benefits the oral administration as it increases the number of contacts with the gastrointestinal mucosa [130]. The ideal average size reported for NPs transcytosis appears to be approximately 100 nm in gastrointestinal applications [130, 131]. This size distribution enables NPs to be transported into the gastrointestinal wall by enterocytes and M cells, which typically take up NPs with diameters ranging between 20 and 100 nm and 100–500 nm, respectively [132]. Rod-shaped NPs constantly outperform spherical particles, a fact consistent with trends indicating that nanorods are absorbed more effectively than spheres into epithelial cells, suggesting that NPs geometry influences in vivo fate [133, 134]. However, only a limited fraction of the NPs taken up by the intestinal epithelial cells undergoes exocytosis [135]. Passive diffusion across the digestive system wall is restricted, even when the said shapes maximize NPs transport. Hence, the active exploration of novel targeting approaches is needed.

The transferrin pathway could be exploited to ease the transepithelial transport into the colon [136]. Targeting techniques in the gastrointestinal tract are hampered by the production of coronas in gastrointestinal fluids, which change with diet, and goblet cells that produce mucus to coat the mucosa surface. Both concerns impose constraints on the interactions of NPs with intestinal walls [137]. The characteristics of these barriers profoundly change due to pathologies such as inflammatory diseases, increasing the epithelial permeability, and affecting mucus production, pH, and gut flora. Thus, the gastrointestinal tract's limitations, compounded by patient pathologies, constitute significant hindrances to obtaining therapeutically desirable NPs biodistributions via oral administration.

### Microenvironmental barrier

Once NPs reach the target location, they must negotiate the local microenvironment. Owing to the complex and continuously evolving microenvironment, the NPs penetration becomes even more difficult. Thus, to properly engineer NPs that reach the targeted tissues or cells, it is necessary to have a fundamental understanding of the microenvironments in which they will work.

#### Microenvironment-associated variability

Generally, tissue microenvironments are strikingly different from those proper of the circulation, thus significantly altering NPs physical characteristics and stability. For example, the gastrointestinal system has regions of severe pH fluctuation, acidity, and the presence of degrading enzymes. These factors make the gastrointestinal system a destabilizing habitat for many NPs [88, 138]. Additionally, disease states can alter the gastrointestinal microenvironments, resulting in a variable reactivity to biomaterials. For instance, an investigation of colon cancer and colitis microenvironments indicated disease-specific compatibility with dendrimer/dextran biomaterials [88]. Variable pH fluctuations occur in different disease-related microenvironments. Low pH levels are found in many tumors, or the variable pH levels are detected throughout the wound healing process [139, 140]. Certain pH-responsive nanocarrier platforms have been designed that enable drug release exclusively under defined pH conditions. Xu et al. demonstrated that Lipid-coated CaCO_3_ NPs as a novel pH-responsive drug delivery platform for the treatment of breast cancer by enabling combined chemotherapy [141]. Zhu et al. present a pH-sensitive nanomedicine that combines an enzyme with focused ultrasound to ablate tumors and to facilitate hypoxia in hypoxia-prone environments to improve the efficacy of DOX-based chemotherapy [142]. Since wound sites are typically hyperthermic, temperature-responsive NPs can adapt to the local environments and offer a customized drug delivery [143]. In the case of atherosclerosis and vascular stenosis, constricted arteries generate increased shear stresses, which can be used to enhance the therapeutics released from NPs that degrade under such conditions [144].

Tumor microenvironments are also crucial as they can influence the NPs fate. NPs can accumulate in tumors due to their extravasation across leaky arterial walls, a process related to an increased permeation and retention (EPR) effect [79]. Some studies correlated the EPR effect to NPs accumulation in tumors, where 10–15% of injected NPs accumulate compared to 0.1% of free drugs [79]. However, in a recent study Sindhwani et al. demonstrated that passive transport, including the EPR effect, accounts for a discrete percentage of NPs accumulation in a rat tumor model. This increased accumulation of NPs in tumors could be due to immune cell interactions, protein coronas, and molecular processes [145].

Limited perfusion is also a barrier to therapeutic NPs delivery into the brain. Because of the restricted extracellular space and non-specific ECM adherence in the brain microenvironment, NPs typically fail to penetrate the tissue after crossing the BBB [146, 147]. To overcome this hindrance, advanced delivery methods have been proposed to improve NPs diffusions, such as convection-enhanced delivery (CED) and surface modifications, including a dense PEG coating. These approaches helped enhance NPs delivery and penetration into glioblastomas [148].

#### Mucus penetration

Biofilms and mucus are two more barriers to the local distribution of NPs (Fig. 3). Within mucus layers, the lengths between adjacent polymer linkages dictate the mesh pore size, ranging between 10 to 1000 nm, allowing for the diffusion of smaller items while trapping bigger ones [149, 150]. Mucus can also trap items via non-specific interactions, allowing their fast removal from epithelial surfaces. Mucus behaves differently depending upon its physiological location due to changes in its composition, hydration, and viscoelasticity [151, 152]. The gastrointestinal mucus forms a thick adhering layer, but the pulmonary mucus is reportedly thinner and more mobile, making it a heterogeneous barrier and preventing drugs from reaching the lungs [151, 152].

**Fig. 4:**
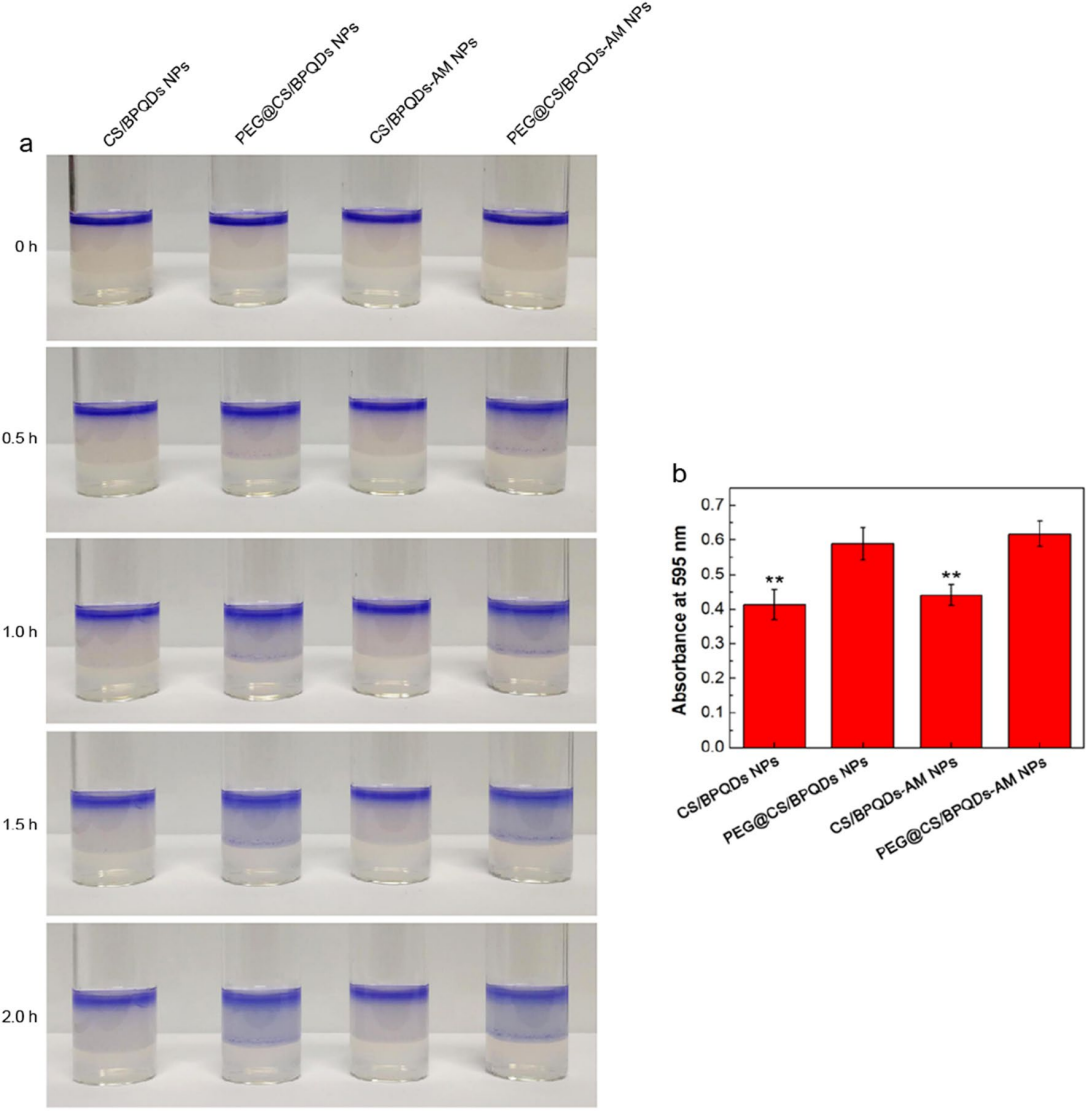
PEG surface modification enhances the ability of BPQDs to penetrate the sputum layer. **A** Visual inspection of how various samples penetrate through the artificial mucus layers. **B** Absorbance (at 595 nm) detection at the bottom layer agarose gel of sputum layer penetration by diverse nanoparticles two h after administration. CS chitosan, AM amikacin, BPQDs black phosphorus quantum dots. Reproduced with permission from Ref. [85] Copyright 2020, John Wiley and Sons

Although the mucus behavior is consistent throughout various physiological contexts, mucus features can vary between distinct regions of an organ system, and all these barriers are adaptive. As an example, mucus thickness and turnover rate in the gastrointestinal tract are affected by fiber intake [151, 152]. A significant pH difference exists between the near-neutral ECs surfaces and the acidic intestinal lumen, making the mucosal barrier a destabilizing environment for NP platforms [88, 152]. The pathology of the gastrointestinal system can alter glycosylation patterns, pH, and mucus layer thickness [153]. Similarly, lung diseases do alter local mucus behavior. Mucus in the lungs significantly affects the absorption of inhaled NPs conveying substantial amounts of MUC5AC and MUC5B polymers [154, 155]. However, elevated MUC5B levels and excessive polymer cross-linking reduced mucus pores size and clearance rates in cystic fibrosis, promoting bacterial growth by entrapping pathogens and restricting neutrophil movement [155, 156]. Mucus attributes vary widely depending on patient characteristics, including ongoing pathology, diet, and lifestyle in the respiratory tree, making it a challenging physiological environment for inhaled NPs delivery. COPD is characterized by a thick layer of mucus, needing a novel method for therapeutic drug delivery, i.e., NPs with mucus-penetrating capabilities. In a recent study, Li et al. demonstrated the drug release from nanovehicles by black phosphorus quantum dots (BPQDs) for efficient therapy of COPD. The PEGylated chitosan (CS) nanospheres were coupled with BPQDs. The hydrophilic PEG and the positive charges of CS enabled the nano-vehicles to deeply penetrate the mucus layer (Fig. 4) [85]. Next, the interior of the BPQDs rapidly degraded into nontoxic PO_4_ and acidic H^+^, facilitating the dissociation of the PEGylated CS nanospheres and eventually promoting the drug's release [85].

### Cellular uptake and intracellular trafficking barriers

Even once NPs have contacted target cells, several barriers to NPs absorption and intracellular trafficking influence their functional cargo delivery (Fig. 3). This section discusses the barriers a nanocarrier must overcome in order to achieve a proper cellular uptake and internal trafficking. The intracellular barrier is the final biological hurdle against the delivery of therapeutic agents. The corona, combined with altered NP properties, including hydrophilicity and charge, affects cellular absorption in macrophages, cancer cells, and numerous other cell types [157, 158]. Cell surfaces are composed of negatively charged phospholipid bilayers that hold biomolecules in a fluid mosaic structure [157, 159]. Human cells have around 400 types of cell surface transporters, with lipid rafts and transmembrane proteins being the most common membrane components [157, 159]. Additionally, the precise rigidity of the cell membrane and its structural fluidity is partly regulated by the cytoskeleton, which dynamically responds to external inputs [160].

Thus, NPs engaging with the same cell type may have distinct interactions based on their cell membrane locations and/or contact time. Due to reciprocally repulsive negative charges, anionic NPs struggle to engage the cell surface. Conversely, cationic NPs with excess positive charges can damage the cell membrane and induce cytotoxic effects [161, 162]. Thus, the first encounter between an NP and a cell may decide the NP's fate and therapeutic potential. Few definite trends about the optimized NPs shape and size have been established regarding cellular uptake. According to several studies and models, spherical NPs outperform rod-shaped NPs in non-phagocytic cells [163], while other studies demonstrate the reverse upshot [164, 165].

**Fig. 5:**
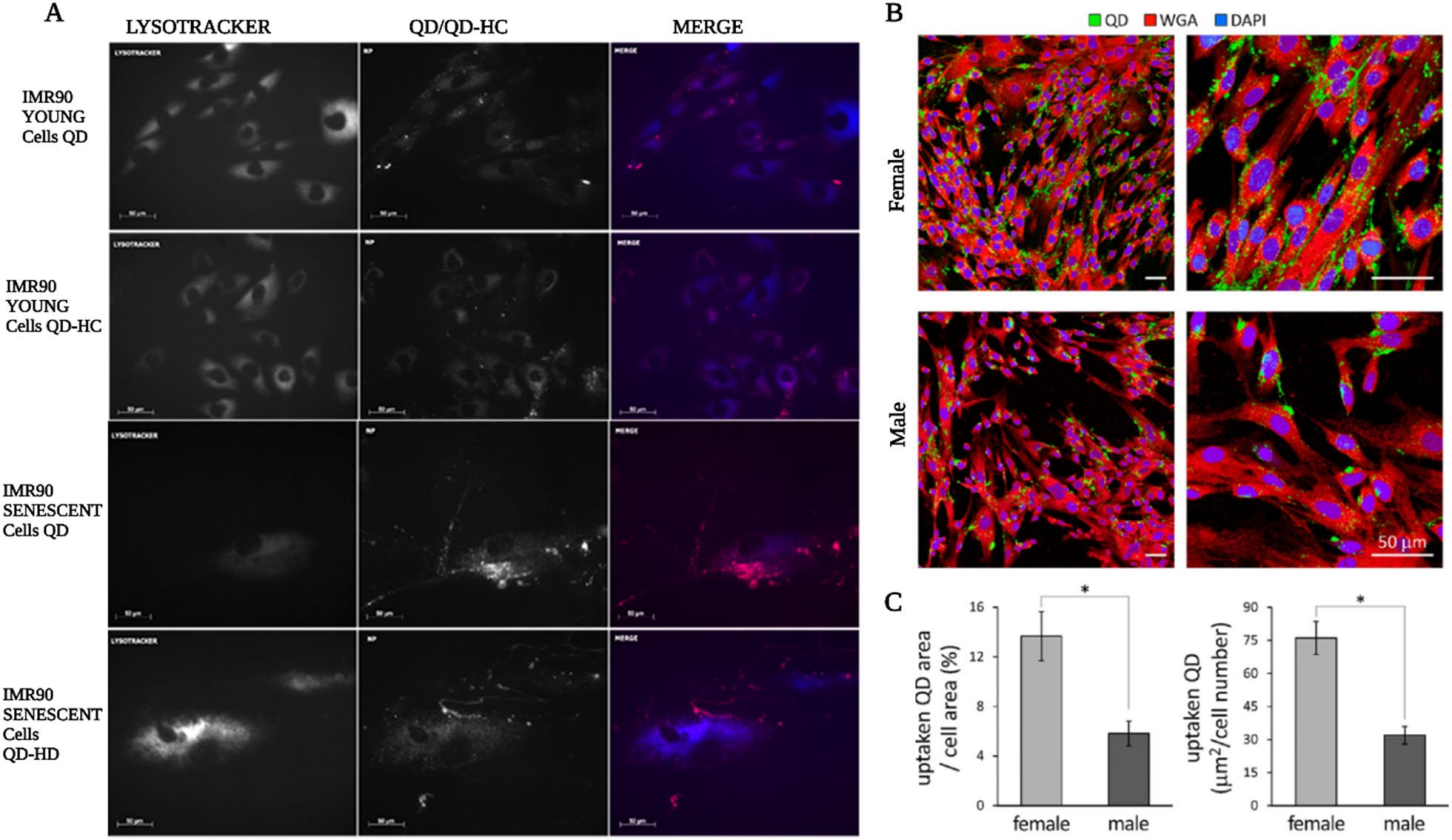
Effect of cell age and sex on uptake of nanoparticles. **A** Fluorescence micrographs of NP colocalization in lysosomes of young IMR90 and senescent IMR90 cells. Experiments were conducted at 37 °C for 2 h. Lysosomes were stained with LysoTracker Blue fluorescent dye (blue) and quantum dots (QD)/QD-HC red marked nanoparticles. Bar = 50 μm. Reproduced with permission from Ref. [168] Copyright 2019, American Chemical Society. **B** Immunohistochemical imaging of female and male human amniotic stem cells (hAMSCs) demonstrated a significant increase in QD uptake in female *vs* male cells. The scale bar represents 50 μm in all panels. (C) QD uptake was quantified using confocal images (*n* = 7/group). Reproduced with permission from Ref. [169] Copyright 2018, American Chemical Society

Similarly, several in vitro studies have proved that non-phagocytic cells only uptake NPs with a diameter of 10–60 nm and that smaller NPs internalize more efficiently. But other researches indicated that smaller NPs are more likely to induce cytotoxicity [3, 89, 166]. The process of NP absorption can be classified as passive or active [49]. Due to the selective permeability of the cell membranes, passive diffusion is mainly confined to smaller, uncharged molecules traveling along concentration gradients [162]. Thus, NPs oftenest penetrate the cell membrane via active transport [4, 89]. However, caveolin-mediated endocytosis can occur in certain interactions with negatively charged NPs [162]. Caveolin-mediated endocytosis occurs when molecules smaller than 60 nm are engulfed and employ lipid rafts to form specialized vesicles [167]. This type of endocytosis is more commonly observed with nanorods than the nanospheres. Clathrin-mediated endocytosis relies on receptor-mediated, electrostatic, or hydrophobic contacts between NPs and the cell membrane in Clathrin-expressing cells [157]. Thus, various parameters decide the mechanism(s) by which NPs are taken up, including the cell membrane characteristics and the capabilities of the NPs, both of which affect the later endocytic process. Many cellular differences also result from an individual's traits. For example, younger human fibroblasts and epithelial cells took up more NPs than older ones, and the younger cells were less affected by toxicity (Fig. 5A) [168].

Additionally, sex-related differences were observed to influence the absorption of AuNPs in the case of human amniotic stem cells and salivary fibroblasts [169], thereby revealing another factor to consider in NP delivery (Fig. 5B). Furthermore, drug-resistant cells add to the cellular heterogeneity that complicates NPs distribution [170]. For example, some cancer cells can develop resistance to platinum (II)-based medications, such as cisplatin and oxaliplatin, which disrupt DNA structure to induce apoptosis.

As a result, smart NP systems must be designed to overcome these challenges. For example, micelles carry NPs more efficiently to the nucleus, reducing the chances of developing drug resistance [171]. Thus, both phenotypes and cell types contribute to the heterogeneity of the cell population, posing a variety of challenges to NP delivery. However, recent advances in NP design may help overcome these constraints.

## Alternative strategies to enhance nanoparticle design

To account for the significant variability of disease states and biological barriers within and between patient groups, strategies for administering therapeutics must be exceedingly modular and adaptable. Nanomedicine and nanodelivery systems can be produced in various ways, which may lead to a greater scientific understanding of their in vivo interactions, facilitate clinical translation, and enable the development of novel therapeutic approaches. The differences in NPs composition and structure make it challenging to predict and control off-site ancillary effects. The latter include inflammation, undesired organ targeting, and sequestration by the MPS. This section discusses the impacts on the delivery of various NP characteristics, emphasizing how specific choices about NP design can overcome barriers tailored to particular diseases.

### Rapid degradation and elimination strategies

A significant barrier to NPs is the drastically distinct metabolic pathways that differentiate them from small-molecule drugs or biologicals. While the pharmacokinetics of these common/conventional medications have been thoroughly studied and proven, NPs often show different behaviors for reasons rooted in their macromolecular structure. Simultaneously, the bulk of smaller organic molecules is removed predictably and efficiently through various metabolic pathways. Conversely, numerous nanomaterials defy rapid and effective clearance from the body. Human biology lacks the metabolic pathways or mechanisms capable of processing significant quantities of nanomaterials or nanoscale particles. Additionally, the proclivity of many NPs to target specific organs, particularly the liver and spleen, enhances the possibility of triggering (potentially deleterious) secondary effects, especially when, after administration, bio-persistent materials loiter for long and possibly cause off-site ancillary effects [172].

Constructing the NP delivery systems from biodegradable nanomaterials is a workable and practical strategy apt for mitigating the secondary impacts of biopersistence. Under physiological settings, biodegradable nanomaterials break down into nontoxic compounds, and their elimination via natural pathways is rapid. For instance, PLGA is a copolymer of polylactic acid and polyglycolic acid that degrades into its two original monomers, lactic acid, and glycolic acid, which are two naturally occurring metabolites in human physiology. Consequently, biodegradable nanomaterials, such as polylactic acid, poly(amidoamine), and chitosan, are widely used [173]. These polymer systems are highly adaptive and versatile and can modulate a condition-specific degradation, such as pH, or systematically regulate their own degradation rate. In vivo, biodegradable NPs used in medical applications are more likely to disintegrate in bursts rather than gradually decay [172]. Even though an NP is biodegradable, its physicochemical features, such as charge, size, and hydrophobicity, may exert undesirable effects on the blood coagulation system. An alternate approach to ease the removal of intravenously delivered NPs is synthesizing ultrasmall (~ 10 nm) NPs with positive surface charges [174]. Ultrasmall NPs filtration by the renal glomeruli and urinary excretion is swift. This approach was clinically tested: inorganic silica-coated C-dots with a diameter of approximately 7 nm were quickly cleared by the kidneys [175, 176]. A disadvantage of ultrasmall particles is that their extremely quick elimination may not suit specific nanomedicines. However, an improvement of this method might be using biodegradable NPs, such as bigger polymeric NPs, that dissolve or dissociate into smaller components the kidneys can clear.

### Harnessing the Corona Towards Nanoparticle Design

The "corona" is a critical factor regulating NPs interactions with the host's biology. Historically, the corona development has been considered as an undesired 'biofouling' that should be prevented, for example, by using low-adhesion NP coatings, such as PEG or other 'stealth' materials, to obscure the particle from immune detection and reduce its biological interactions. However, it is unlikely that the corona formation, which affects the NPs biological outcome, will ever be fully eliminated. A more forward-thinking approach to nanotherapeutics would be promising strategies that may result in a better scientific knowledge of NPs interactions in vivo to develop innovative treatments. The corona should be viewed as an NP's extension rather than an obstacle. Its development and composition should be studied and analyzed, considering the particle's intended purpose. One may use the NP's immaculate identity as a foundation to construct its biological profile. Thus, altering the corona architecture can alleviate issues associated with nanotherapeutics, including biodistribution, cytotoxicity, immunogenicity, and intracellular compartmentalization. In order to enhance the in vivo biological behavior, NP surfaces are modified with chemical agents or coated with various proteins and lipids [79, 177, 178]. It is possible to control the NP's biomedical behavior by controlling material parameters. Corona preformation is a simple practice by which, before their administration, NPs are preincubated ex vivo in a predetermined medium with the desired corona components. Corona preformation enables some degree of control over the corona makeup and can be applied to all the currently available NP types. Although this technique was studied in animal models primarily using albumin as the sole corona constituent, this unsophisticated and rather simple method has demonstrated its potential to reduce the binding of opsonin and complement immune factors while improving NPs stability and decreasing particle cytotoxicity [179, 180]. More sophisticated preconfigured coronas can be developed with a greater understanding of nano-bio interactions, such as easing NPs targeting or reducing off-target ancillary effects associated with NP biopathology. However, since the NP's corona is a dynamic shell [181], this approach has limitations and drawbacks. Although a preformed corona may play a role in deciding an NP's first biological identity, it is a temporary structure susceptible to altering the host's biological environment.

Another more sophisticated and advanced method to harness the corona is to use an in-silico modeling technique for rational particle design, allowing to forecast and direct corona development in vivo. The software modeling molecular dynamics and structure–activity relationships could predict corona assembly and NP-cell interactions [182, 183]. More modeling could analyze how corona-bearing NPs interact with biological barriers and their behavior in biological systems. These approaches could be combined into a rational particle design. The NP's physicochemical properties, shape, and functionalization should be devised to enable the assembly of a corona of the desired composition, i.e., suitable for the NP's biological function.

Similarly, related research showed that manipulating the polysaccharide chain structure outside a dextran NP can activate certain complement immune pathways [184]. The NP surface can be functionalized to attract specific components into the corona. Thus, an AuNPs functionalization motif was designed in silico to include transferrin from blood serum into the corona. Additionally, a recent study of NP surface chemistry showed that it is possible to influence the conformation of the bound corona components. In fact, polymeric NPs endowed with distinct surface functional groups could stabilize or denature the corona's proteinaceous albumin [185].

Altogether, these investigations have revealed that it is possible to 'fine tune' the NP's corona to minimize undesirable off-site ancillary effects. However, none of these experimental methodologies is exhaustive. Hence, a combination of diverse analytical approaches is needed to predict the in vivo biological outcomes and the practical application of the coronas in pharmaceutical sciences. Such as, it is necessary to perform a thorough assessment of the NP-protein interaction under-stimulated biomimetic conditions [186, 187]. It is especially critical to carefully set up media exposure factors such as proteins, fluidic shear stress, and degradable enzymes. Furthermore, new pharmaceutical technologies like tumor-on-a-chip models and biomimetic microfluidic systems allow for a deeper examination of NP behavior [188]. However, the practical barriers and insufficient information about the nano-bio interface should be addressed. To be effective, both the preformed corona method and the rational NP design strategy require a much deeper understanding of corona dynamics and biological interactions.

### Advanced biocompatible and biomimetic NPs

A particle's biocompatibility corresponds to its potential to avoid causing unpleasant reactions in the host biology, as many of such adverse reactions are products of undesirable interactions with nanomaterials. Obviously, any clinical application requires a high degree of biocompatibility. However, NPs materials of frequent clinical use, such as PEGylated NPs, may be less biocompatible than previously assumed. Many investigations have discovered a significant incidence of anti-PEG antibodies in patient sera [93, 189], showing that PEGylation is relatively immunogenic. As a result, we must perfect and enhance currently available nanomaterials. Fundamental elements of pristine identity, including physicochemical properties, shape, geometry, and density, significantly affect NPs biocompatibility and thus should be tailored to meet different requirements and help modulate the Nano-Bio interactions. For example, it is known that deformable disc-shaped and hemispherical polymeric particles outperform rigid and spherical particles in terms of biocompatibility, possibly because they mimic the shape, size, elasticity, and surface tension proper of erythrocytes [190].

Apart from employing synthetic nanomaterials, another strategy for improving biocompatibility is incorporating biomaterials into the NP design. This approach could be beneficial by mitigating adverse off-site ancillary effects associated with NPs, as endogenous materials are far less likely to elicit unpleasant reactions. The NP can be masked with a biomimetic exterior by cloaking it in native material using natural biomolecules.

Another method is to conjugate the NPs surface to cell membrane/platelet-derived vesicles, or even to whole cells, to effectively 'hitchhike' the NPs and conceal them via the associated cell membrane, as is the case of the NPs conjugated with erythrocytes [191, 192]. However, this approach might trigger unintended NP-induced effects on the carrier erythrocytes and thus requires the refinement of NPs [193]. Encapsulating the NPs into cell-membrane-derived microvesicles generated from the host's own collected cells is another innovative variation of this approach [194]. The microvesicle-enveloped NPs have an exterior identical to and indistinguishable from any other endogenous vesicle. In this situation, the NPs are encased in a cell-derived membranous 'ghost' that degrades upon uptake into the targeted tissue to expose the NP's core.

**Fig. 6:**
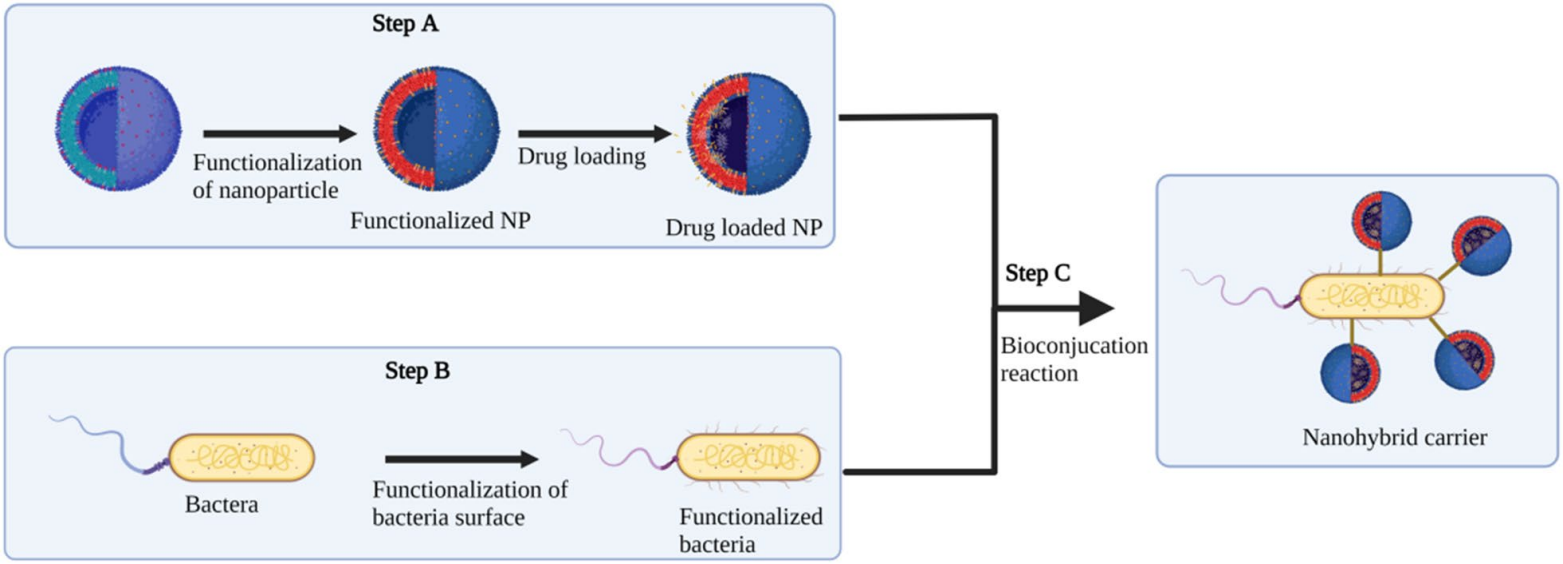
The general scheme of bioconjugation reactions for the synthesis of drug-loaded nanobiohybrid carriers. Step A: NPs are first functionalized with a chemical partner and then loaded with the therapeutic agent of choice. Step B: The chemical group is also incorporated into the surface of bacteria. Step C: Bioconjugation reaction between the chemical partners leads to the bioconjugate bacteria-nanomaterial

Additionally, a whole core NP can be designed from bioidentical chemicals naturally found in the host biology. This approach might be used with a variety of biomaterials, including lipoprotein-like particles, membrane-derived microvesicles, and virus-like particles (Fig. 6). By using material that is compatible with the host's own biology, such techniques may further mitigate any unexpected outcomes associated with foreign nanomaterials or hypersensitivity responses.

### Exosome-based drug delivery

Bioinspired nanoscale drug delivery systems comprised of exosomes or small extracellular vesicles (EVs) have gained much attention in the past two decades. The human body contains innate vesicles known as exosomes found in nearly all cells. Therefore, there has been tremendous progress in understanding exosomes, including their mechanisms, function, and compositions. Recently, considerable research has been done on modifying the exosomes, encapsulating therapeutic payloads, and in vivo disposition [195, 196]. Significant developments have been made toward optimizing exosome delivery to solid tumors [197–199]. Exosome bioengineering involves the development of targeted exosomes for treatment to increase target specificity by overexpressing naturally existing surface proteins or fusing a targeting ligand with exosome-enriched transmembrane proteins [200, 201]. Increasing the specificity between the molecules targeting recipient cells will improve therapeutic efficacy and reduce side effects. In particular, there is an emphasis on exosomes as endogenous NPs capable of crossing various biological barriers, which may represent the beginning of a revolutionary era in medicine, with their applications in prevention, intervention, and a range of imaging, drug and gene therapies. Clinical translation of synthetic NPs has been limited, but exosomes may be a viable alternative in delivering therapeutic substances with enhanced specificity, bioavailability, and reduced risks. In recent years, increasing information has emerged about EVs to manipulate them to serve as specialized drug delivery systems [196, 202, 203].

There has been extensive research conducted on the BBB, yet it remains one of the most difficult barriers to overcome. The composition of tight junctions delimits the main boundaries of the BBB and reduces permeability. Recent studies have demonstrated that exosomes can cross the BBB due to their specific homing properties, immunogenicity, and extended half-life [204, 205], the first step towards a more endogenous approach to this complex dilemma. Despite this, the role played by exosomes in barrier integrity has largely remained unclear. It has been demonstrated that exosomes can carry cargo, including miR-3p, across the BBB [154]; to alleviate inflammation in hemorrhage-affected areas in the brain and deliver anticancer agents that specifically target neurons [204] and oligodendrocytes.

Additionally, they can be taken up by the brain parenchyma and other brain cells [206]. Moreover, exosomes carry miR-132, which has been identified as a regulator of adherens junction-related proteins, which leads to increased BBB permeability [207]. Recent research has shown that exosomes derived from brain stem cells may trigger and repair BBB breakdown, reducing and reversing BBB-induced Alzheimer’s disease [45]. Such crucial capabilities of exosomes in maintaining the integrity of the BBB imply an ever-increasing therapeutic significance.

Numerous studies have demonstrated the efficacy of using exosomes as a delivery system for therapeutic agents to cancer cells. Munagala et al. worked on transferring drugs to lung tumor cells by utilizing the space of exosomes and its advantages in terms of drug loading [208]. The results showed that the drug could provide potential remedial effects in the mice model. Similarly, Morishita et al. induced specific responses by using exosomes derived from murine B16BL6 melanoma cell lines [209]. Furthermore, it was determined in a previous study that when exosomes were used to deliver DOX, the negative effect of the drug on the heart was significantly reduced, in addition to inhibiting breast cancer tumor growth [210]. Furthermore, cancer suppressor compounds need to be properly correlated with the carrier and exosome in order to achieve a good therapeutic effect.

The studies of exosomes have become increasingly popular in the field of nanotechnology. Several challenges are associated with relying on specific surface molecules to deliver therapeutic agents to specific cell types. Several of the exosomal components are yet to be identified or characterized, and their interaction and interference with other cells in the host cells remain unclear. Nevertheless, many quality attributes that determine the tissue distribution, the cell type-specific uptake, and the intracellular drug release of the administered exosomes, in addition to spatiotemporal information regarding exosome fate in vivo require more investigation.

### Bacteria as a transport vehicle

The ongoing development of nanomaterials for various diseases has significantly impacted biomedical research. However, nanomaterials' insufficient drug delivery capability to penetrate the diseased area limits their efficiency against a specific disease. The use of bacteria and other microorganisms in conjugation with other nanomaterials has recently acquired popularity due to various advantages over chemical synthesis methods, including low cost, low toxicity, biocompatibility, and ease of synthesis [211]. Because of their flagella, some bacterial species can self-propel and guide themselves. Chemical reactions involving bioconjugation allow for the facile attachment of NPs into living systems, such as bacteria or cells. As a result, bacteria are employed as a delivery vehicle for nanomaterials, allowing easier penetration and subsequent drug release (Fig. 6).

Therapeutic agents or NPs [143] must be distributed evenly throughout the tumor to successfully affect the total tumor cell population. Still, this scenario does not occur often, and particles accumulate in the tumor margins' perivascular regions [212]. This results in tumor locations with low drug concentrations, which supports the sprouting of quiescent cells that are mainly resistant to chemotherapeutic treatment. As a result, it is critical that antitumoral therapy explicitly targets the deeper and hypoxic regions of the tumor for optimum treatment. Due to bacteria's self-propulsion and guidance capabilities, bacteria-mediated tumor therapy (BMTT) allows for successful intratumoral targeting [213]. It is possible for them to actively swim away from the tumor vasculature and reach more favorable settings for bacteria growth in deeper locations. Flagellated bacteria's intrinsic motility permits them to permeate tissues regardless of hydrodynamic concerns [214]. Diverse bio-hybrid nanocarriers made of a wide range of nanomaterials have been discovered since BMTT emerged as a new technique for efficiently penetrating and combating tumors. These nanocarriers have been used to carry therapeutic drugs, genes, or proteins, to deep tumor locations inaccessible to conventional chemotherapy. However, the results vary depending on the use of nanomaterials.

Similarly, bacteria-mediated nanomaterials can effectively penetrate the sputum barrier in inhalation injury. Airway burning or inhalation injury forms the thick viscous sputum barrier and results in respiratory obstruction and severe pulmonary infection. In this case, the airway mucus gel layer is a critical barrier to successful drug delivery [215]. The sputum penetrable bacteria-mediated nanomaterials could hopefully address these issues by using nanotechnology. The introduction of genetically modified facultative anaerobic bacteria, such as *Salmonella* or *E. coli* could be a viable solution to the problem of NPs penetration. The broad therapeutic toolbox that genetically modified microorganisms imply can be used in conjunction with innovative clinical tactics to remodel disease therapies into something more effective and safe.

### Carrier-Free Nanomedicines Delivery Systems

**Fig. 7:**
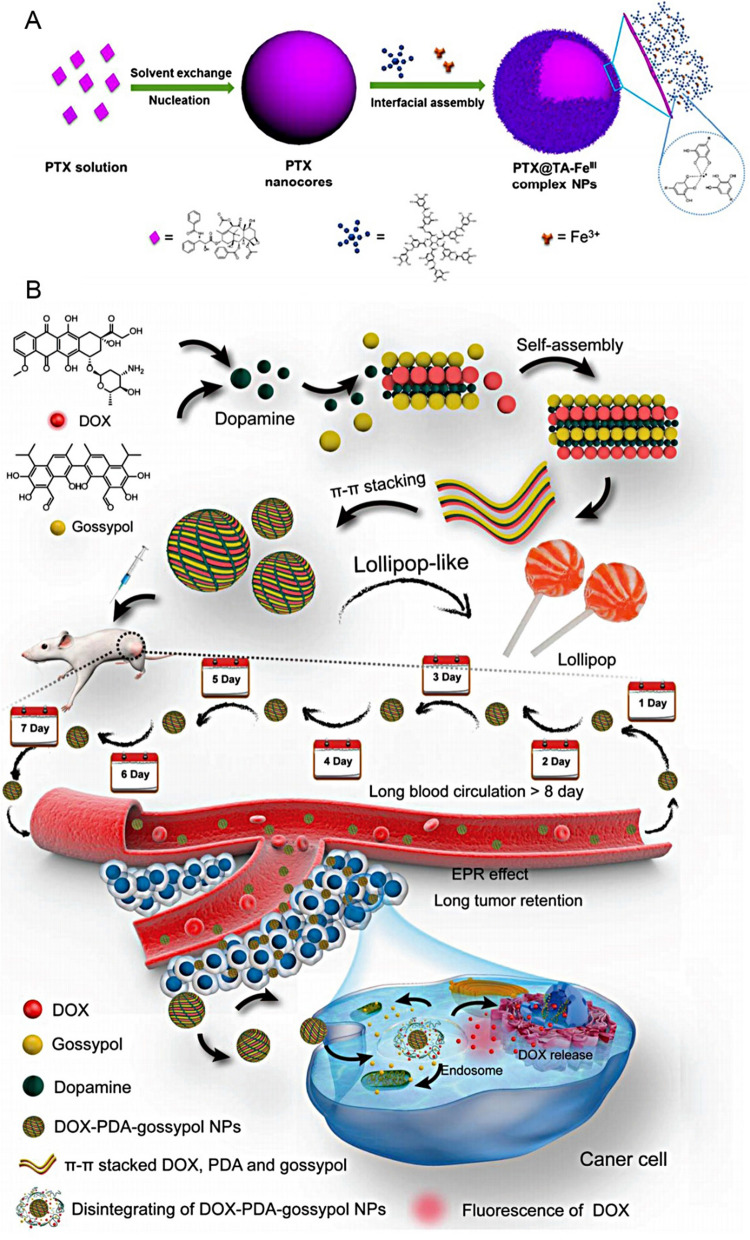
Strategies for carrier-free nanodrugs to improve drug delivery and stability. **A** A bioinspired coating offers a novel method to successfully inhibit and delay the Ostwald ripening of hydrophobic drug nanoparticles, resulting in smaller and evenly sized particles that provide enhanced drug delivery and long-term colloidal stability. Reproduced with permission from Ref. [221] Copyright 2016, American Chemical Society. **B** A novel strategy to prepare lollipop-like dual-drug-loaded nanoparticles, Polydopamine (PDA), fills the gaps between the doxorubicin (DOX) and gossypol molecules to form the super-compact long-circulating nanoparticles and enhanced tumor penetration. Reproduced with permission from Ref. [222] Copyright 2019, John Wiley and Sons

A fundamental limitation of nanomedicine carriers is their limited drug-carrying capacity (about 10% wt/v), which hinders the accumulation of effective drug dosages and their therapeutic effects [1]. In this respect, carrier-free nanoagents have made considerable progress due to their ease of synthesis, high drug loading capacity, and ability to function as an "all-in-one" platform (Fig. 7). However, native defects restrict their precise delivery. Any excessive treatment of chemicals during the preparatory phase can cause serious side effects to the body, significantly impairing therapeutic efficiency and jeopardizing their further development [216].

Numerous efforts to perfect the physicochemical features of carrier-free nanodrugs have improved their delivery and release behavior. However, studies have proven that the in vivo complex microenvironment and diverse biological barriers can critically determine their therapeutic efficacy [78, 217]. The proper adjustments of the drug ratio in carrier-free nanoagents have enabled the precise control of the NPs' size, shape, and surface charges, contributing significantly to their in vivo fate, including blood circulation time and drug release. Various strategies are adopted to address these issues, including the surface modification of the NPs with different polymers, small molecules, or proteins to boost their stability without impairing their drug loading capability (Fig. 7). While current strategies can help mitigate some of the concerns associated with an early drug release, significant issues still need to be addressed by future investigations.

## The future of nanomedicine

Nanomedicine is one of the most exciting areas of research today. In recent decades, extensive research in this field has already led to the filing of numerous patents and several dozen clinical trials. Nanomedicine and the application of nano-drug delivery systems provide precise delivery of a drug to the affected cell type, such as cancer and tumor cells, without affecting the physiology of normal cells. This trend will continue to be a major topic of research and development for many decades to come.

NP platforms provide a variety of customizable characteristics, including shape, size, charge, surface qualities, and responsiveness, that may be chosen to optimize delivery for a particular application, treatment, and patient group. Customization of NP platforms may improve patient stratification methods when screening NP platforms, broaden patient access to precision therapeutics by allowing new patients to qualify for existing therapies via enhanced delivery mechanisms, and ultimately increase precision medicine and NP platform effectiveness. Design considerations have become more complex and sophisticated, as have efforts to generalize patterns across large groups, compromising the precision of the findings within a small population to develop a general principle, an all-encompassing principle of delivery.

To improve the specificity of these claims, it is important to thoroughly analyze all aspects of NP design and their interactions with the human body, particularly as we move toward stratifying patient populations to establish the most appropriate NP platforms for each subgroup. For example, the concept of controlled drug release at the beleaguered sites has not yet been completely perfected. We should focus our efforts on developing materials with increased uniformity and the ability to load and release the drug. Researchers have the opportunity to collect data and study outcomes from the continued exploration of nanotechnology in laboratory settings, which will add to the ever-growing library of design-function relationships known in nanomedicine. Nevertheless, it is crucial to contextualize the trends observed in research settings before generalizing findings. Since minor variations in NP composition, animal models, and pathologies can substantially impact NP performance and must be addressed when advancing NP technology towards clinical translation.

NPs have achieved successful clinical outcomes largely in the diagnostic sphere, such as recognizing early stages of disease using specific ligand-receptor interactions or biomarkers to determine which therapeutics are best suited for a specific patient. For instance, assessing the degree of the EPR effect shown by a cancer patient might indicate the efficacy of NP treatment accumulation at the solid tumor site [218]. However, these platforms have enormous potential for improving the efficacy of precision medicine therapies. Potential future applications of metal-based NPs such as gold, silver, and magnetic NPs in diagnosis and therapy could advance the development and use of nanomedicines on a larger scale. For example, AuNPs have garnered a lot of attention, as they appear to be readily absorbed by soft tumor tissues and render the tumor more susceptible to radiation (e.g., in the near-infrared)-based heat therapy for selective elimination [219, 220]. Most of these materials are biocompatible and stable, and they fulfill specialized applications that need qualities that organic materials cannot provide. However, there is a need for continuous innovation, and currently, the technology is in the infancy stage of research. In addition, scientists and researchers should also emphasize the immune response in living organisms rather than concentrating solely on developing nanomaterials that have similar structures to native tissues.

Besides, restricting the number of patients eligible for specific NP-based therapeutics might reduce the potential market size. At the same time, the cost of NP-based therapeutics could be high if only a specific population group could administer them. However, NP platforms that are effective in certain patient populations may have the potential to administer various precision-based and generic therapeutics. Thus, constructing a single NP platform effective for a stratified population might result in several successful therapeutic applications. Moreover, precision NP designs may allow for better therapeutic efficacy than NPs produced for wide populations, and considerable gains in survival, quality of life, and even dosage might justify the increased cost of these precision delivery systems.

## Conclusions

This review has explored several conceptual NP engineering designs for the successful administration of therapeutics and their tailoring to overcome the various biological barriers met across patients suffering from diverse diseases. Such barriers to proper drug delivery are worsened by patients' comorbidities, disease development phases, and specific tissues physiology. NP platforms can be customized in terms of shape, size, charge, and surface qualities to maximize delivery for a particular application, treatment, and patient group. These NP features have been researched across several biological conditions, and in certain situations, exploitable trends have been validated for intelligent NP design. For instance, the charge is crucial to muco-penetrating NPs and intracellular applications that need an escape from endosomes. Applications requiring cell types to absorb NPs gain precedence over-targeting surface markers, such as in many cancers and therapeutic applications. These breakthroughs in NP design entail enormous potential to increase the effectiveness of Precision Medicine therapies, but diagnostic applications have yet to see clinical developments. This lack of clinical advancements is mainly due to evaluating the efficacy of NP platforms in large populations. The considerable variability in biological barriers found in high numbers of patients may obscure the possibility of successfully treating smaller subgroups. Thus, further explorations into NPs design and later interactions inside the human body are necessary to increase the precision of these claims, particularly as we move toward stratifying patient populations to set up the most proper NP platforms for each subgroup.

## Abbreviations

NPs: Nanoparticles
FDA: Food and Drug Administration
LBNPs: Lipid-based nanoparticles
LNPs: Lipid nanoparticles
PNPs: Polymeric nanoparticles
PDMS: Poly(dimethylsiloxane)
PEG: Poly(ethylene glycol)
PAMAM: Polyamidoamine
AuNPs: Gold nanoparticles
IONPs: Iron oxide nanoparticles
ENP: Engineered nanoparticles
RES: Reticuloendothelial system
MPS: Mononuclear phagocytic system
ECs: Endothelial cells
CNS: Central nervous system
VCAM: Vascular cell adhesion molecule
COPD: Chronic obstructive pulmonary disease
CS: Chitosan
BPQDs: Black phosphorus quantum dots
BBB: Blood-brain barrier
BMTT: Bacteria-mediated tumor therapy
